# Pharmacokinetic studies of *hyperoside*-2-hydroxypropyl-β-cyclodextrin inclusion complex and ameliorated DSS-induced colitis in mice

**DOI:** 10.1042/BSR20230003

**Published:** 2023-05-18

**Authors:** Jianqing Su, Xinyu Zhang, Shengliang Cao, Cheng Liu, Xiang Fu, Rui Zhang, Xiaoli Li, Jiaojiao Xue, Ying Li, Xueyan Wang, Yi Ding, Yubao Li, Xiuling Chu

**Affiliations:** Agricultural Science and Engineering School, Liaocheng University, Liaocheng 252000, China

**Keywords:** 2-hydroxypropyl-β-cyclodextrin, colitis, Hyperoside, intestinal flora, pharmacokinetics

## Abstract

An inclusion complex formation with cyclodextrin is a promising method to improve the bioavailability of water-insoluble drugs. The pharmacokinetic characteristics of *Hyperoside*-2-hydroxypropyl-β-cyclodextrin inclusion complex in rats were evaluated. Compared with *Hyperoside*, the results showed that maximum plasma concentration and AUC_0-t_ indexes of *Hyperoside* inclusion complex in rat plasma were increased, the value of half-life time was prolonged, and the value of apparent clearance was decreased, which proved that *Hyperoside* complexed with 2-hydroxypropyl-β-cyclodextrin could improve its bioavailability and increase its blood concentration. Secondly, the therapeutic effect of *Hyperoside* before and after complexing was further compared through the dextran sodium sulfate-induced colitis in mice. The experimental results showed that under the same dose, the *Hyperoside* inclusion complex had a better therapeutic effect, which could significantly increase the body weight of mice, improve the disease activity index, alleviate colon shortening, improve pathological colon changes, and have a better protective effect on colitis mice. According to 16S rDNA sequencing analyses, *Hyperoside*-2-hydroxypropyl-β-cyclodextrin may have an anti-inflammatory effect by increasing the abundance of beneficial bacteria (e.g. Firmicuria) and decreasing the proportion of harmful bacteria (e.g. Bacteroidetes) to balance the colon’s microbiota.

## Introduction

**Figure 1:**
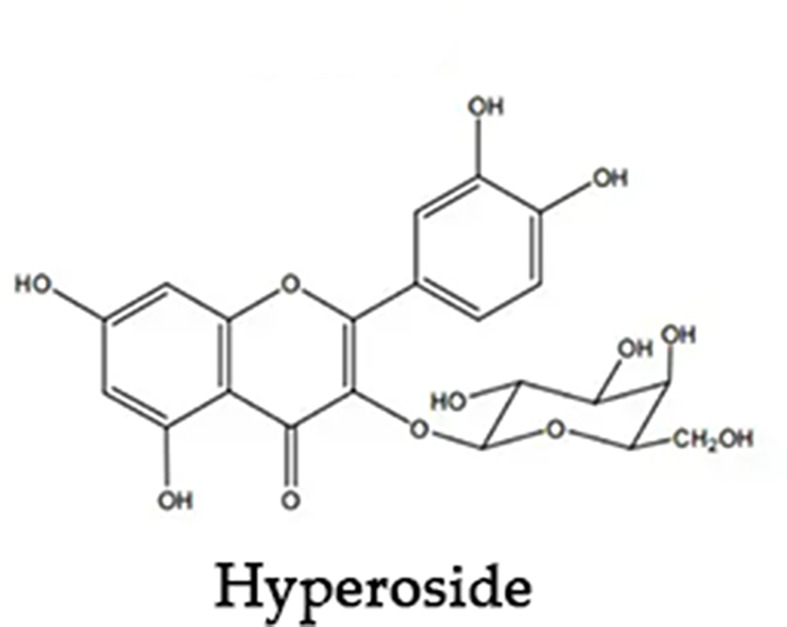
Chemical structure of *Hyperoside*

*Hyperoside* (Hyp) is a kind of natural flavonols glycoside that is widely found in many medicinal plants such as *Hypericaceae*, *Rosaceae*, *Leguminosae*, and *Celastraceae*. Its chemical structure is shown in Figure 1. Hyp has anti-oxidative stress [1,2], anti-inflammatory [3], anti-apoptosis [4], and other biological activities [5]. Oral administration is the most common route of drug administration [6]. However, Hyp is an insoluble substance with poor solubility in water [7], poor oral absorption, and low bioavailability, which greatly affect its clinical application [8]. Previous research results confirmed that the absorbed site of Hyp was small intestines in the gastrointestinal tract [9]. The study found that the pharmacokinetic characteristics of oral administration were short time to peak and absolute oral bioavailability was only 1.2%, indicating poor oral absorption [10].

An inclusion complex formation with cyclodextrin is a crucial method to solve insoluble drugs [11]. Through the use of a range of non-covalent contacts, including van der Waals forces, hydrogen bonding, and hydrophobic interactions, cyclodextrins complexed pharmaceuticals to create water-soluble host-guest cyclodextrin polymers [12], thereby increasing drug solubility [13], bioavailability [14], stability [15], and pharmacological activity [16,17]. Arya et al. [18] used β-cyclodextrin and curcumin to form an inclusion complex, which increased its water solubility by 206 times. Duarte et al. [19] prepared the inclusion complex of methyl-β-cyclodextrin and resveratrol, which increased the water solubility of resveratrol by 400 times and retained the potent antioxidant activity and antibacterial activity of resveratrol and the ability to inhibit the activity of Caco-2 cells. According to the previous research results of the research group [20], Hyp was complexed with 2-hydroxypropyl-β-cyclodextrin (2H-β-CD) at a molar ratio of 1:1 to create the inclusion complex. The solubility of Hyp in water was significantly increased by 9 times. The thermal stability and antioxidant effect of Hyp were improved after complexing (*P*<0.05), but the bioavailability and clinical efficacy of *Hyperoside*-2-hydroxypropyl-β-cyclodextrin (Hyp-2H-β-CD) have not been reported. Generally, the bioavailability of the inclusion complex will increase due to the improvement of solubility, and then the effectiveness will be enhanced. Hsu et al. [21] investigated the protective effect of angelica-β-hydroxypropyl-cyclodextrin complex on CCl_4_-induced liver failure in mice,and the findings revealed that the inclusion complex more effectively decreased the contents in aspartate aminotransferase, alanine transferase, and liver malondialdehyde than angelica extract. Buko et al. [22] evaluated treatment effect of 2-hydroxypropyl-β-cyclodextrin with sertraline inclusion complex in the rats induced by alloxan, and the results showed that inclusion complex significantly reduced the severity of diabetes, decreased blood glucose and glycated hemoglobin levels, and regulated serum insulin levels and insulin sensitivity return to normal compared with clathrate.

Antioxidant and anti-inflammatory activities are significant pharmacological effects of Hyp [1]. The gut is usually a frequent site of oxidative stress and inflammation [23]. Hyp has been shown to guard against enteritis by increasing the activity of antioxidant enzymes and lowering the generation of peroxides [24,25]. Colitis is often used as an antioxidant and anti-inflammatory model for drug evaluation [26]. To further verify that this new formulation strategy can better improve the bioavailability of Hyp, the pharmacokinetic characteristics of Hyp and Hyp-2H-β-CD inclusion complex were compared by high-performance liquid chromatography (HPLC) in rats. At the same time, a dextran sodium sulfate (DSS)-induced colitis mouse model was built [27], and the therapeutic effects of Hyp-2H-β-CD were compared with that of Hyp. The 16S high-throughput V3-V4 region sequencing analyses of intestinal flora were used to elucidate the therapeutic mechanism. This provides ideas and an experimental foundation for the research and development of an excellent new dosage formulation for oral administration.

## Materials and methods

### Animals care

The 48 Specefic pathogen Free (SPF) male Institute of Cancer Research (ICR) mice, aged 6–8 weeks, weighing (22 ± 2 g), and 9 SPF male Sprague Dawley (SD) rats, weighing (250 ± 20 g) were obtained from Jinan Pengyue Experimental Animal Center. The mice were raised in an animal room free of specific pathogens in College of Agriculture, Liaocheng University. The temperature was controlled at (24 ± 2°C) and (50 ± 5%) relative humidity with a 12 h light–dark cycle. Before the experiment, these animals were acclimated to the lab environment for a week before being starved for 12 h. During the investigation, they were guaranteed to drink water and eat freely. All animal experiments were carried out in strict accordance with the recommendations in the Guide for the Care and Use of Laboratory Animals. According to the guidelines, ether was used to euthanize the mice. The Liaocheng University Animal Ethics Committee approved the protocols for animal studies (Permit Number: 20200126).

### Reagents and instruments

*Hyperoside* (HPLC grade) and *Baicalin* (Bai) were provided by Yuanye Biotechnology Co., LTD. (Shanghai, China), the Sodium carboxymethyl cellulose, methanol (AR ≥ 99.9%), phosphoric acid (85–90%), and 2-hydroxypropyl-β-cyclodextrin were purchased from McLean Biochemical Co., LTD. (Shanghai, China). DSS was bought from Pomerania Biotechnology Co., LTD. (Hefei, China). Baori Physical Science and Technology Co., LTD. (Beijing, China) provided the Agarose gel DNA extraction kit.

Ultrasound instrument (Power-Sonic SB-600DTY; Xinzhi Biotechnology Co., LTD., Ningbo, China), freeze dryer (LGJ-10; Songyuan Huaxing Biotechnology Co., LTD., Beijing, China), constant temperature oscillator (HZQ-F160A; Yiheng Scientific Instrument Co., LTD., Shanghai, China), table high-speed refrigerated centrifuge (Microfuge22R; Beckman Coulter Co., LTD., California, U.S.A.), chromatograph (waters 2695; Waters Technology Co., LTD., Shanghai, China), Ultra-fast liquid chromatograph (LC-30AD; Shimadzu Enterprise Management (China) Co., LTD., Shanghai, China), PCR instrument (GeneAmp®9700 model; Applied Biosystems Inc., U.S.A.), and 12 tube water bath nitrogen blower (HSC-12A; Shanghai Reunion Scientific Instrument Co., LTD., Shanghai, China).

### Method

#### Preparation of Hyp-2H-β-CD

According to the method previously studied [20], the inclusion complex was prepared according to the molar ratio of 1:1. An appropriate amount of *Hyperoside* powder was dissolved in absolute ethanol, and an appropriate amount of 2H-β-CD was dissolved in water by stirring in proportion. Then the Hyp ethanol solution was added drop by drop into the stirred 2H-β-CD solution. The homogeneous and saturated inclusion complex solution was obtained by ultrasonic mixing for 1 h and then shaking for 72 h. After cooling, 0.2 μm cellulose membrane was used to remove the undissolved solids, and the filtrate was first frozen in a refrigerator at −80°C, and then transferred to a freeze dryer for lyophilization to obtain the yellow Hyp-2H-β-CD inclusion complex, which was used for later experiments. The solubility of Hyp-2H-β-CD in water increased to 1351.24 µg·ml^−1^, nearly nine times more than the solubility of Hyp (153.09 µg·ml^−1^).

When administered to animals, appropriate amounts of Hyp and Hyp-2H-β-CD inclusion complex were weighed and prepared with distilled water to form oral preparations of Hyp and Hyp-2H-β-CD inclusion complex, which were dispersed by ultrasound, and the concentration of preparation was adjusted according to the body weight of mice. Sodium carboxymethyl cellulose (CMC-Na) was used to prepare 1 g·L^−1^ suspension of Hyp. The administration mode was intragastric administration.

#### Pharmacokinetic test

##### Dosing protocol and blood collection

Three SD rats were placed in each of the three groups, which were chosen at random. Before gavage, the rats went without water for a 12-h fast. The rats in the Hyp group (20 mg·kg^−1^), Hyp-2H-β-CD group (20 mg·kg^−1^), and H-Hyp-2H-β-CD group (40 mg·kg^−1^) were administrated according to the gavage volume, respectively. The rats were given a regular diet and water 4 h after intragastric administration. At 0 h before administration and at 0. 08, 0. 25, 0. 50, 0. 75, 1, 2, 3, 4, 6, 8, and 12 h after administration, heparinized capillary tubes were used to draw 0.3 ml of blood into heparinized EP tubes from the fundus venous plexus of rats under ether anesthesia. The samples were centrifuged for 10 min at a speed of 825 RCF (g), and the top plasma was collected and kept at −80°C pending testing.

The treatment procedure was conducted according to Cui [28] and Liu [29] et al. Approximately 100 μl of plasma was taken into a tube, then adding 10 μl of the internal standard solution (baicalin, 50 μg·ml^−1^), and vortex mixing for 1 min. After adding 1.0 ml of ethyl acetate, the liquid was well mixed using a 3 min vortex before being centrifuged at 6740 RCF (***g***) for 10 min at 4°C. The residue from the upper layer of ethyl acetate was redissolved in 100 μl of mobile-phase methanol after being transferred to another clean centrifuge tube and dried with nitrogen. The supernatant layer was removed following further centrifugation at 9705 RCF (***g***) for 10 min (4°C) and kept there until testing. Blood was taken from rats before administration as blank plasma, and an internal standard solution was not added in the treatment of blank plasma samples. The remaining steps were performed as described above.

##### HPLC method

The HPLC method was established, and the pharmacokinetic method and results are shown in the Appendix 1.

#### Animal experiments

**Figure 2:**
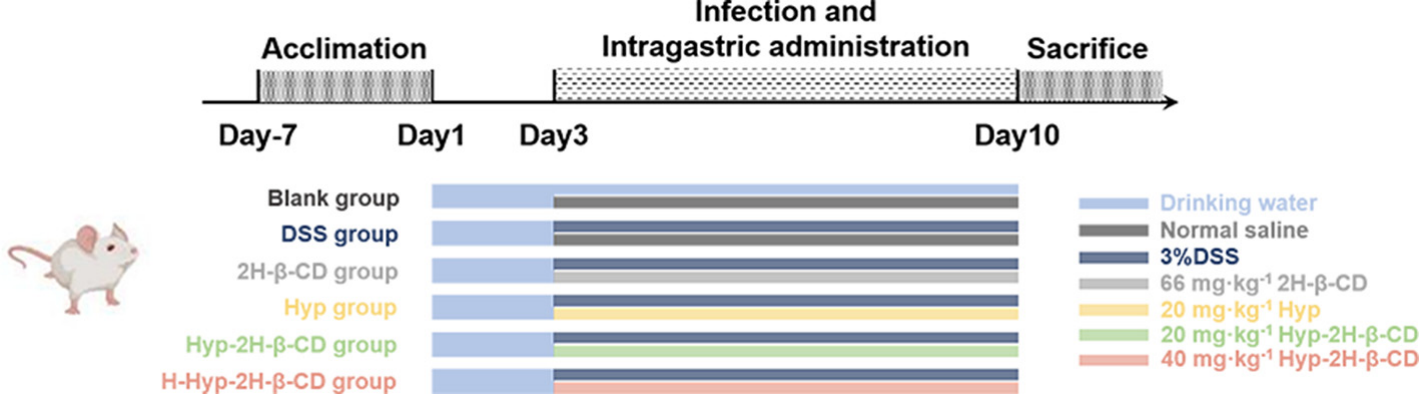
Experimental design

We divided the SPF ICR mice into six groups at random. The mice were divided into blank group, model group (DSS group), cyclodextrin group (2H-β-CD group), Hyp group (Hyp group), Hyp-2H-β-CD low dose group (Hyp-2H-β-CD group), and Hyp-2H-β-CD high dose group (H-Hyp-2H-β-CD group). According to Wirtz [30] and other research methods, except for the blank group, the mouse colitis model was established by drinking 3% DSS solution (0.03 g·ml^−1^) every day. In contrast, the blank group kept the regular water intake. The 2H-β-CD group received 0.2 ml of the 2H-β-CD solution via gavage at a dose of 66 mg·kg^−1^ body weight during the period, while the blank group and the model group received 0.2 ml of normal saline, respectively. Each group received 0.2 ml (Hyp suspension or Hyp-2H-β-CD aqueous solution) via gavage at a dose of 20 mg·kg^−1^ of body weight for the Hyp group and Hyp-2H-β-CD group, respectively. The H-Hyp-2H-β-CD group received 0.2 ml of Hyp-2H-β-CD aqueous solution at 40 mg·kg^−1^ of body weight. The test was conducted over 7 days, and Figure 2 depicts the entire process.

#### Test sample collection

(1) During the trial, the mice's body weight, mental state, hair color, water, food intake, and disease activity index (DAI) score were all recorded daily. DAI was graded using the following standards: (a) score for weight loss (0 = no loss, 1 = 1–5%, 2 = 6–10%, 3 = 11–20%, and 4 = >20%); (b) diarrhea (0 = normal, 2 = loose stools, 4 = watery diarrhea); (c) Blood in the stool (0 points = no bleeding, 2 points = mild bleeding, 4 points = gross bleeding). (2) The experimental mice were fasted 7 days after administration, and at 12 h, the mice were weighed and sacrificed by neck removal. To determine organ index and gauge colon length, mouse heart, liver, spleen, lung, kidney, small intestine, and colon were removed, cleaned with normal saline, and weighed. (3) Colonic feces were collected, and the intestinal segment about 3–4 cm from the anus was cut along the longitudinal axis of the mesentery. To sequence the 16S rDNA of the intestinal flora, feces were collected and kept in sterile frozen tubes in liquid nitrogen. (4) Colonic mucosa gross morphological damage index (CMDI): After the colon segment was cleaned with sterile phosphate buffer saline (PBS) (137 mmol·L^−1^ NaCl, 2.7 mmol·L^−1^ KCl, 4.3 mmol·L^−1^ Na_2_HPO_4_, 1.4 mmol·L^−1^ KH_2_PO_4_, pH 7.2), the colonic mucosa morphology was observed under a magnifying glass, and the damage score was given according to the colonic mucosa gross morphological damage index (CMDI) standard [31] (Table 1). (5) Pathological staining: 1 cm of each colon at the same site was sectioned and fixed with 4% paraformaldehyde. Hematoxylin–Eosin (HE) and periodic acid–Schiff (PAS) staining were used to color the colons after embedding them in paraffin. The pathological changes of the colon were analyzed. Three sections were made for each part of the colon, and the most representative was selected for further investigation. Eight relatively intact villus and eight crypts were selected and measured for each tissue section using ImageJ software. The tissue pathology was graded, and the villus height (VH) to crypt depth (CD) ratio was computed [32] according to Table 2 Under a microscope, the morphology and quantity of goblet cells (GC) in the crypts of 8 PAS diseased sections were examined and quantified.

**Table 1:** CMDI scoring criteria

Score	Colon tissue damage		
0	No hyperemia, edema	Regular folds, clear veins	No ulceration
1	Congestion, edema	No erosion	No ulceration
2		Moderate erosion	No ulceration
3			Single ulceration
4		Severe erosion	multiple ulceration
5			Ulceration > 1cm

**Table 2:** The histopathology score of colonics

Score	Colon tissue damage			
	Percentage of tissue damage /%	Degree of tissue damage	Degree of inflammation	Degree of crypt damage
0	0	None		
1	< 25	Mucosal	Slight	Damage1/3
2	< 50	Submucosal	Moderate damage	Damage 2/3
3	< 75	Muscularis	Severely	Disappeared completely

#### Detection of intestinal microbiota diversity

Hexadecyltrimethy Ammonium Bromide/Sodium dodecyl sulfate (CTAB/SDS) method was used to extract the entire genome’s DNA. The V3-V4 hypervariable region of the bacterial 16S rDNA gene was amplified using the primers 338F (5′-ACTCCTACGGGAGGCAGCA-3**′**) and 806R (5**′**-GGACTACNNGGGTATCTAAT-3′) [33]. Each PCR sample’s amplicons were standardized to equimolar amounts [34]. The assay was scaled to the sequencing needed for each sample using the QuantiFluor™-ST blue fluorescence quantification instrument from Promega. Following the manufacturer’s instructions, sequencing libraries were created using the NEB Next Ultra DNA Library Prep Kit for Illumina (NEB, U.S.A.), and index codes were added. On an Illumina MiSeq instrument, the library was finally sequenced to produce paired-end readings of 250 bp/300 bp.

### Statistical analysis

The primary pharmacokinetic parameters and relative bioavailability were computed after the data were processed using the non-atrioventricular model in DAS 2.0 pharmacokinetic software. The results were given as $\bar{\text{x}}\pm \text{SD}$. For multiple comparisons and significance analysis, SPSS 21 was utilized. Statistical methods: one-way ANOVA (LSD). OriginPro 2021 software was used for mapping. Gut microbiota diversity data analysis was applied using a software platform (https://cloud.majorbio.com). And some gut microbiota diversity data analysis was examined using the Wilcoxon rank-sum test.

## Results

### Pharmacokinetic results

**Figure 3:**
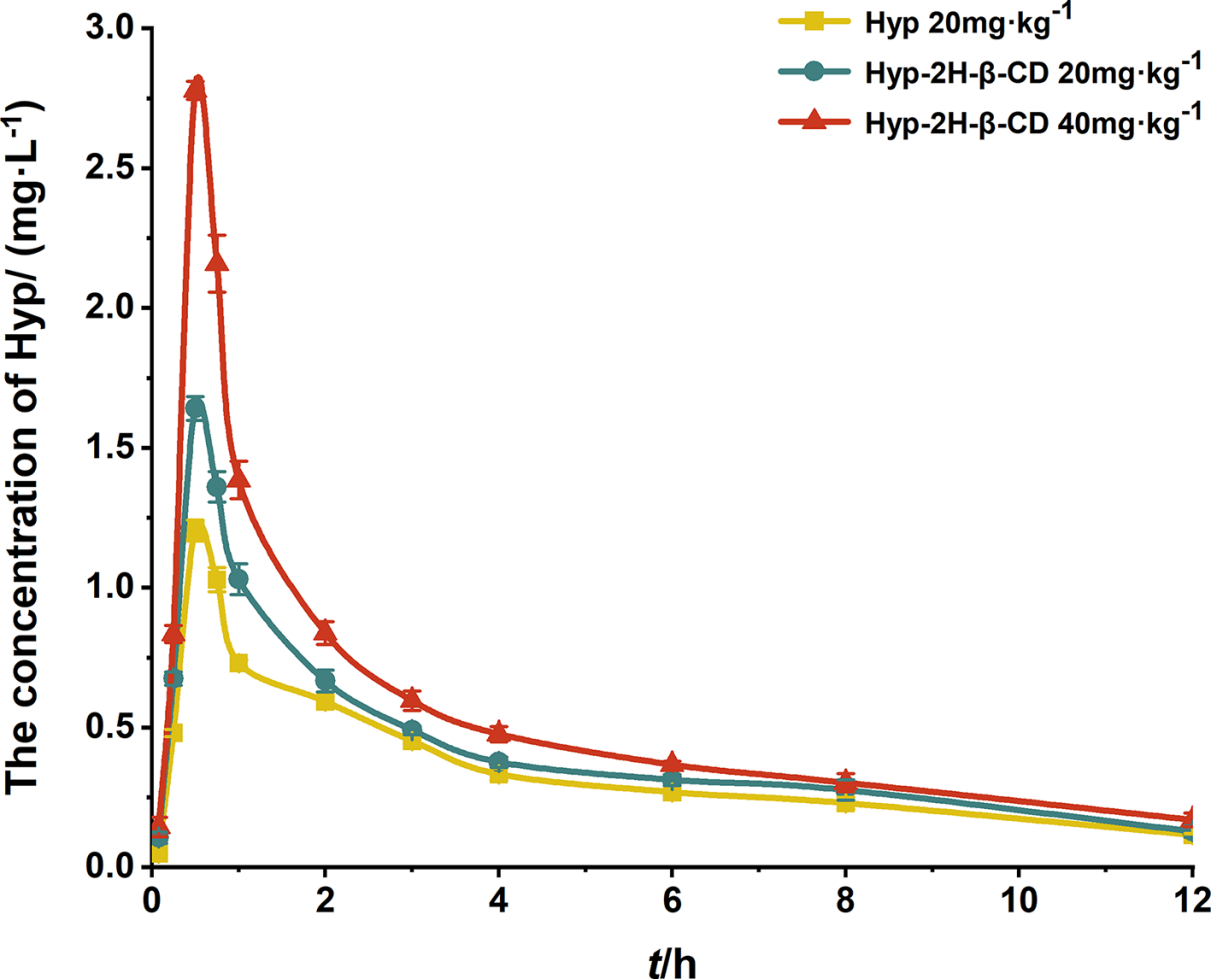
Duration curves of hyperoside after gavage administration of Hyp and Hyp-2H-β-CD (*n*=3)

As illustrated in Figure 3, the blood concentration-time curve was created by calculating the blood concentration at each instant. The findings showed that Hyp-2H-β-CD group had a considerably greater peak plasma concentration than Hyp group after an equivalent dose (20 mg·kg^−1^). DAS 2.0 pharmacokinetic software package automatically fitted the data, and the drug absorption after gavage was in line with the two-compartment model. The relevant pharmacokinetic statistical moment parameters were calculated using the non-atrioventricular model (Table 3). The maximum plasma concentration (*C*_max_) of the isodose Hyp-2H-β-CD group increased from (1.204 ± 0.038) mg·L^−1^ to (1.641 ± 0.042) mg·L^−1^ (*P*<0.0001) and AUC_0-t_ increased from (4.118 ± 0.144) mg·L^−1^·h^−1^ to (4.975 ± 0.192) mg·L^−1^·h^−1^ (*P*<0.01) compared with the Hyp group, indicating that cyclodextrin inclusion complex increased the peak concentration of Hyp *in vivo*, and could significantly promote intestinal absorption of Hyp. When half-life time (*t*_1/2_) was extended from (5.527 ± 1.074) h to (6.286 ± 1.945) h, the apparent clearance (CL/F) value decreased slightly, indicating that the retention time of inclusion complex *in vivo* was significantly prolonged, which showed a certain sustained release effect and increased the recycling time of Hyp *in vivo*. The apparent volume of distribution (V/F) value dropped, but there was no discernible difference, indicating that the two compounds’ *in vivo* distribution ranges were comparable. Following this formula, the relative bioavailability of Hyp-2H-β-CD was determined. Compared with Hyp group, the relative bioavailability of Hyp-2H-β-CD group at the same dose was (120.96 ± 6.26) %. It was speculated that Hyp was complexed with 2H-β-CD, which restricted the entry of Hyp and was not easy to be degraded directly, thus increasing the amount of Hyp through biofilms. Thus, the bioavailability *F* was effectively improved.

$$F=\frac{AUC_{T}}{AUC_{R}}\times \frac{D_{R}}{D_{T}}\times 100\%$$

**Table 3:** Pharmacokinetic parameters of the non-atrioventricular model after gavage administration of Hyp and Hyp-2H-β-CD (*n*=3)

Parameters	Hyp	Hyp-2H-β-CD	
	20 mg·kg^−1^	20 mg·kg^−1^	40 mg·kg^−1^
AUC0-t/mg·L^−1^·h^−1^	4.118 ± 0.144	4.975 ± 0.192*	6.415 ± 0.037†
AUC0-∞/mg·L^−1^·h^−1^	5.123 ± 0.261	6.509 ± 0.988	7.738 ± 0.376*
Cmax/mg·L^−1^	1.204 ± 0.038	1.641 ± 0.042†	2.778 ± 0.032†
tmax/h	0.50	0.50	0.50
t1/2/h	5.527 ± 1.074	6.286 ± 1.945	5.280 ± 0.810
CL/F/L·kg^−1^·h^−1^	3.914 ± 0.204	3.146 ± 0.483	5.182 ± 0.260*
V/F/L/kg	30.954 ± 4.694	27.196 ± 4.446	39.176 ± 4.286

Note: Compared with Hyp group, *P<0.01, †*P*<0.0001.

Where AUC_T_ and D_T_ were AUC_0-t_ and administration dose of test preparation by gavage, AUC_R_ and D_R_ were AUC_0-t_ and administration dose of the reference preparation, respectively.

### Protective effect of Hyp-2H-β-CD on DSS-induced colitis in mice

#### Treatment Effect of Hyp-2H-β-CD against DSS-induced colitis mice

**Figure 4:**
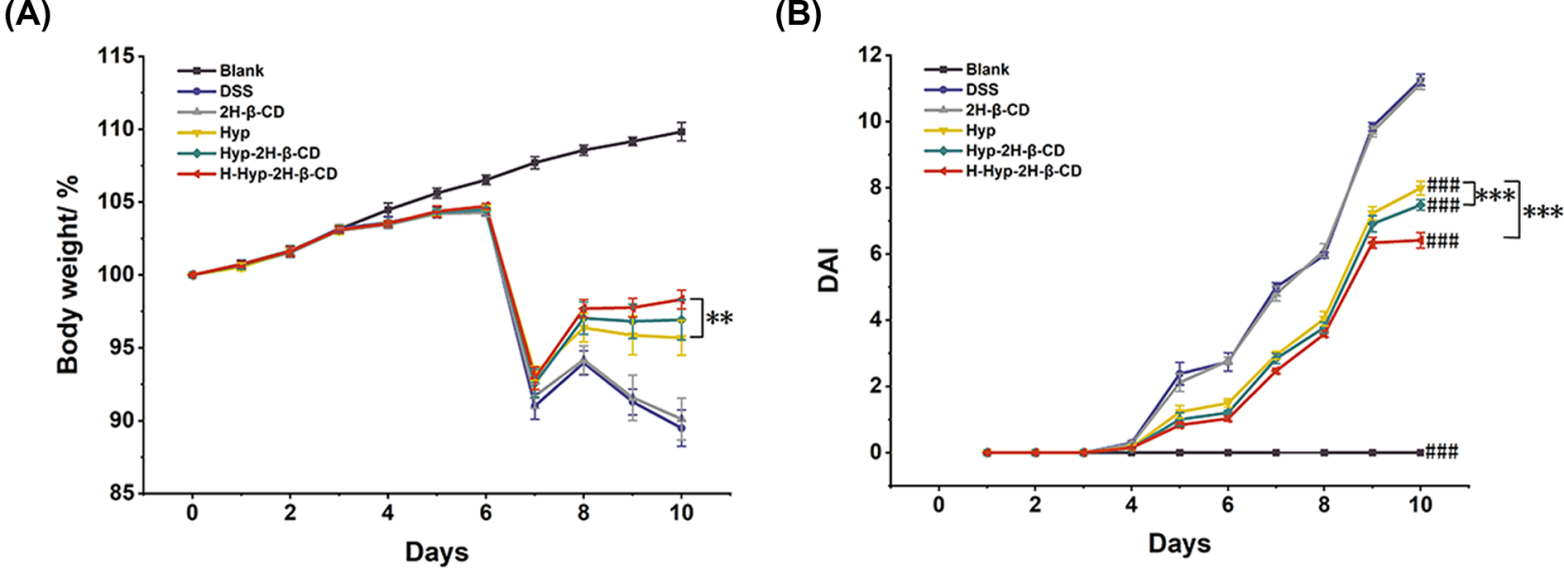
Hyp-2H-β-CD ameliorated the disease phenotype in colitis in mice induced by DSS (**A**) The graph of body weight changes in each group during the experiment. (**B**) Changes trend of DAI score of mice in each group. Note: In figure (**A**) and (**B**), a comparison between two groups, **P<0.01, ***P<0.0001, compared with DSS group, ^###^*P*<0.0001.

The characteristic symptoms of DSS-induced colitis in mice were bloody diarrhea, weight loss, and colon shortening. Figure 4A displays the changes in the weight of the mice in each group. The mice in the blank group grew normally throughout the experiment. However, the weights of the other groups gradually increased after DSS within the first 3 days and sharply declined after day 4 of medication treatment. It was considered that the dose of DSS in mice had reached the onset dose. The body weight of the treatment group's mice increased marginally from day 5 to day 7, but the H-Hyp-2H-β-CD group's mice increased the most, reaching 98.31% of their original body weight, which was substantially greater than that of the Hyp group (*P*<0.01). But it did not reach the recorded value of body weight observed before the test, while the mice in the DSS group’s body weight and 2H-β-CD group fluctuated but still showed a downward trend.

DAI score is an essential index for comprehensively evaluating the degree of ulcerative colitis in mice. The score results are shown in Figure 4B. The fur, mental state, and feeding conditions of mice in the blank group were normal, there was no diarrhea, and the DAI score was close to 0. On the second day of the experiment, the mental state of the other groups was slightly worse, and diarrhea occurred in the DSS group and the 2H-β-CD group. On the 4th day of DSS treatment, mice in the DSS group and 2H-β-CD group showed symptoms such as decreased activity, hair disorder, and blood stool visible by eye, etc. In the administration group, different degrees of blood stool were also detected by a fecal occult blood test, and DAI scores continued to increase. After the Hyp-2H-β-CD injection, the mice gradually recovered after the experiment, and diarrhea and hematochezia were significantly improved. The DAI scores of the two inclusion groups were notably lower than those of the Hyp group (*P*<0.0001). Furthermore, the results of the medication administration group were lower than those of the DSS group (*P*<0.0001), demonstrating that Hyp had a therapeutic impact on mice with colitis. But the effect was stronger after 2H-β-CD complexing, while 2H-β-CD itself had no therapeutic effect.

#### Effect of Hyp-2H-β-CD on colon length and organ index

DSS-fed mice usually showed organ hyperemia, hypertrophy, and other pathological phenomena, as well as the physiological function of damaged organs, decreased, which was manifested by the increase in the organ index value. As shown in Table 4, the spleen index of the mice in the DSS group and 2H-β-CD group was considerably greater than that in the control group (*P*<0.01), and the organ indexes of the heart, liver, lung, and kidney rose. However, there was no statistically significant difference (*P*>0.05). Every organ’s index in the administration group was lower than that of every organ in the DSS group, and every organ’s index in the Hyp-2H-β-CD group was lower than that of every organ in the Hyp group. However, there was no discernible change, and all organ indexes—aside from the spleen—were roughly at the same level as the control group.

**Table 4:** Six-group mice’s organ index

Group	Heart index/%	Liver index/%	Spleen index/%	Lung index/%	Kidney index/%
Blank	0.60 ± 0.12A	5.12 ± 0.37A	0.43 ± 0.09B	0.65 ± 0.11A	1.49 ± 0.08A
DSS	0.74 ± 0.20A	5.34 ± 0.90A	0.80 ± 0.16A	0.72 ± 0.08A	1.66 ± 0.12A
2H-β-CD	0.72 ± 0.21A	5.44 ± 0.48A	0.81 ± 0.20A	0.71 ± 0.07A	1.62 ± 0.11A
Hyp	0.67 ± 0.12A	5.12 ± 0.25A	0.60 ± 0.11AB	0.68 ± 0.08A	1.56 ± 0.11A
Hyp-2H-β-CD	0.60 ± 0.09A	5.01 ± 0.38A	0.57 ± 0.17AB	0.67 ± 0.09A	1.53 ± 0.13A
H-Hyp-2H-β-CD	0.56 ± 0.05A	4.96 ± 0.60A	0.54 ± 0.08AB	0.65 ± 0.15A	1.51 ± 0.11A

Note: In the same column, the different letter means significantly different (*P*<0.05), while those with the same letter mean not significantly different (*P*>0.05).

**Figure 5:**
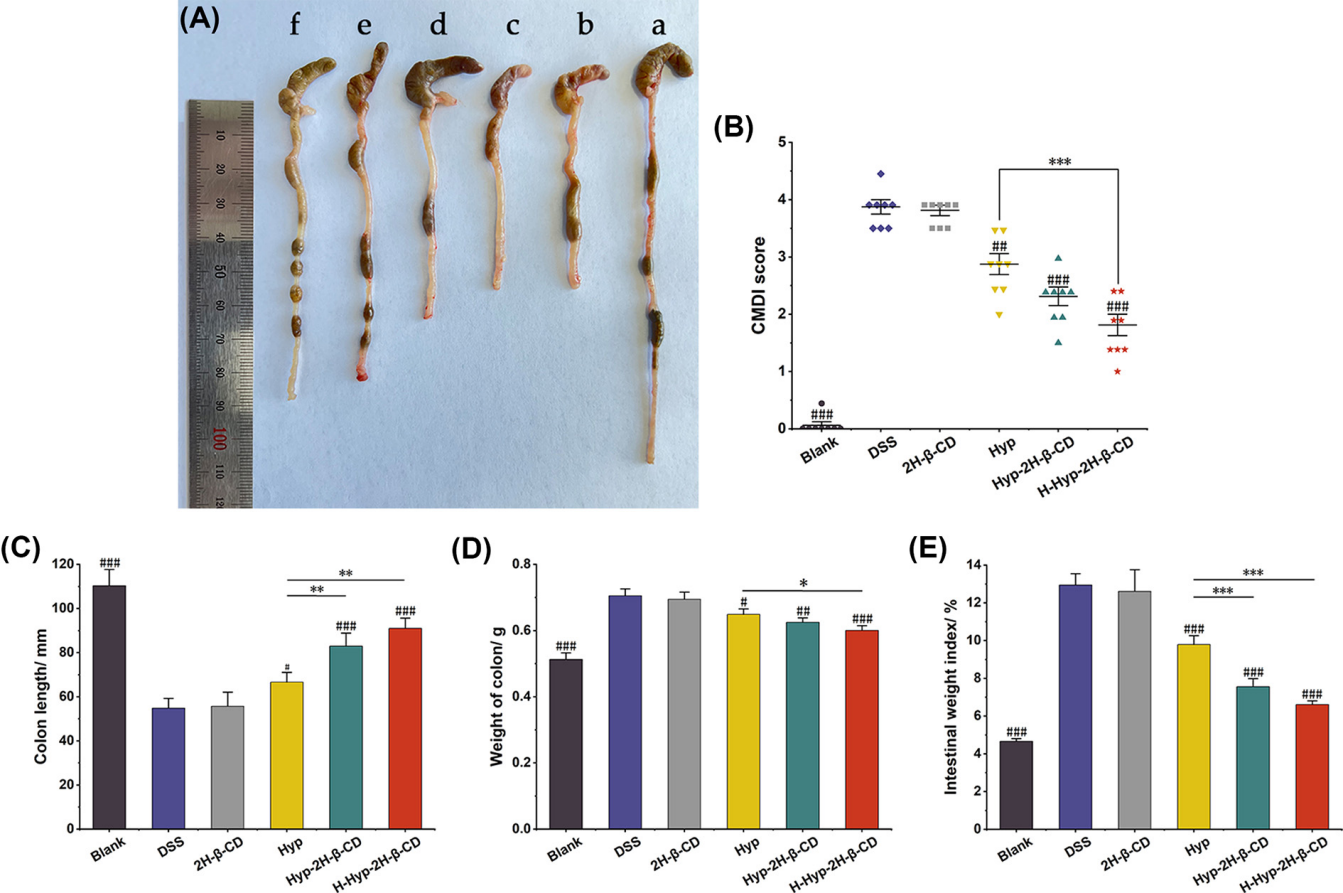
Hyp-2H-β-CD treatment attenuated DSS-challenged colitis in mice (**A**) Comparison diagram of the colonic length of mice in each group. (**B**) Effects of Hyp and Hyp-HE-β-CD on the CMDI score of colonic mucosae in colitis mice. (**C**) Determination results of the colonic length of mice in each group. (**D**) Determination results of the colonic weight of mice in each group. (**E**) Calculation results of intestinal weight index in each group. Legend Description: A, Blank group; B, DSS group; C, 2H-β-CD group; D, Hyp group; E, Hyp-2H-β-CD group; F, H-Hyp-2H-β-CD group. Note: In Figure (B–E), a comparison between two groups, *P<0.05; **P<0.01; ***P<0.0001; compared with DSS group, ^#^*P*<0.05; ^##^*P*<0.01; ^###^*P*<0.0001.

In addition to the increase in the spleen index, the shortening of the colon is another sign of the severity of colitis [35]. In mice in the DSS group, the colon length was greatly reduced, the colon weight was significantly raised, and the intestinal weight index was significantly elevated compared to the blank group (*P*<0.0001), as illustrated in Figure 5A,C–E. The colons of mice in this group were dissected longitudinally along the mesentery. Under the magnifying glass, the intestinal mucosa was observed to be significantly hyperemia and edema accompanied by extensive erosion and ulceration. The CMDI score was substantially higher than the score for the control group (*P*<0.0001, Figure 5B). Compared with the DSS group, colon length was increased, colon weight was decreased, and intestinal weight index was significantly decreased (*P*<0.0001). The effect of Hyp-2H-β-CD group inhibited colon shortening (*P*<0.01) and decreased intestinal weight index (*P*<0.0001) was more obvious than that of the Hyp group. Colonic mucosal tissue in the group experienced a marked reduction in hyperemia and edema. The inflammatory substances essentially disappeared, there were fewer ulcers and erosions, and the CMDI score was much lower than that of the Hyp group (*P*<0.0001). These results suggest that Hyp can improve colon shortening, intestinal injury, and splenomegaly more effectively after 2H-β-CD inclusion in colitis mice. The higher the dose of Hyp-2H-β-CD, the stronger the protective effect on colitis mice.

#### Colonic tissue Morphological in mice with DSS-induced colitis

**Figure 6:**
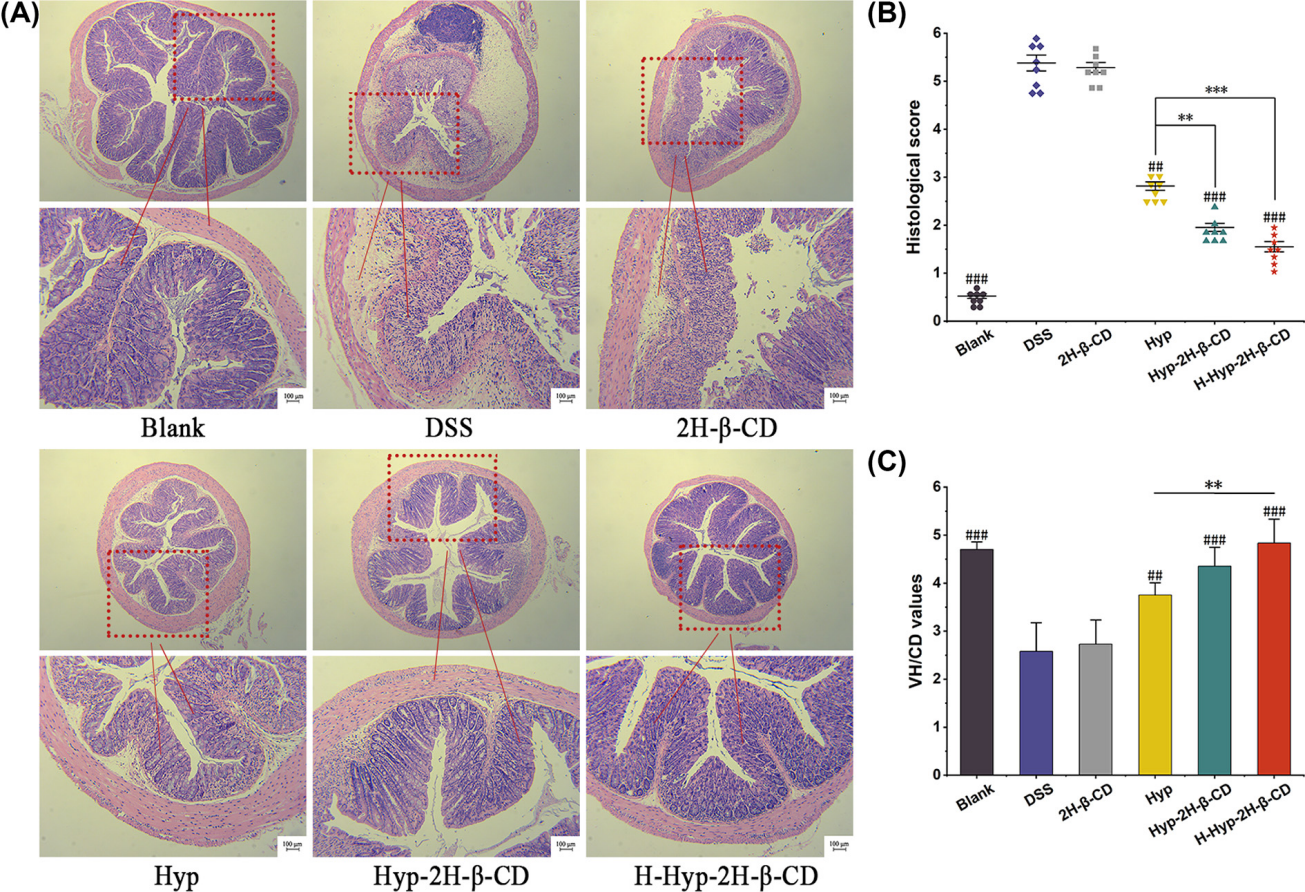
Effect of Hyp-2H-β-CD on colonic lesions in colitis mice (**A**) Colonic histopathological sections of mice from each group (HE staining, ×40 and ×200 magnifications). (**B**) Colonic villus length and crypt ratio of mice in each group. (**C**) Colonic histopathological scores of mice in each group. Note: In Figure (B) and (C), a comparison between two groups, **P<0.01; ***P<0.0001; compared with DSS group, ^##^*P*<0.01; ^###^*P*<0.0001.

The colon and intestinal tract of the mice in the control group were unaltered, and under the microscope, there were no indications of inflammatory infiltration. As shown in Figure 6A, the villi and glands were nicely aligned, and the crypts were apparent. Mice in the DSS group had less intestinal mucosal integrity. The mucosa and submucosa were heavily infiltrated by inflammatory cells, small intestinal villi were lost, intestinal adenolysis and crypt structure were entirely lost, and the histopathological score was higher than that of the control group (*P*<0.0001), as shown in Figure 6B. The mice in the 2H-β-CD group suffered identical colonic damage to that of the DSS group. Still, both Hyp and Hyp-2H-β-CD could improve the histological changes caused by inflammation to different degrees. Although Hyp had a therapeutic effect, part of inflammatory infiltration could still be observed. The therapeutic effect of Hyp-2H-β-CD was more obvious than that of Hyp, and mucosal injury was less severe. Inflammatory cell infiltration was less, goblet cells and their crypts were complete, and histopathological scores were significantly reduced (*P*<0.01). The H-Hyp-2H-β-CD group had the best effect on colitis, with clear villi and crypt structure, lamina propria glands restored to tubular shape, intestinal gland secretion hyperfunction, and colon structure returned to normal. The histopathological score of the H-Hyp-2H-β-CD group was the lowest (except for the blank group), which contrasted markedly with those of the Hyp group (*P*<0.0001). Additionally, the capacity for digestion and absorption can be somewhat predicted by the relationship between villus height (VH) and crypt depth (CD). When the villus became shorter, or the crypt deepened, the value of VH/CD decreased, indicating the presence of intestinal mucosa inflammation. Results in Figure 6C show the ratios of the DSS group and 2H-β-CD group were 54.78% and 57.96% of the blank group, respectively. The findings were regarded as being highly noteworthy (*P*<0.0001), indicating that DSS induced intestinal inflammation in mice, and 2H-β-CD had no therapeutic effect once more. Compared with the DSS group, the ratio of the Hyp group, Hyp-2H-β-CD group, and H-Hyp-2H-β-CD group increased stepwise, and there was a significant difference between the H-Hyp-2H-β-CD group and Hyp group (*P*<0.01). These findings suggested that clathrate, when used dose-dependently, could mitigate the morphological colon damage brought on the DSS-induced mice.

**Figure 7:**
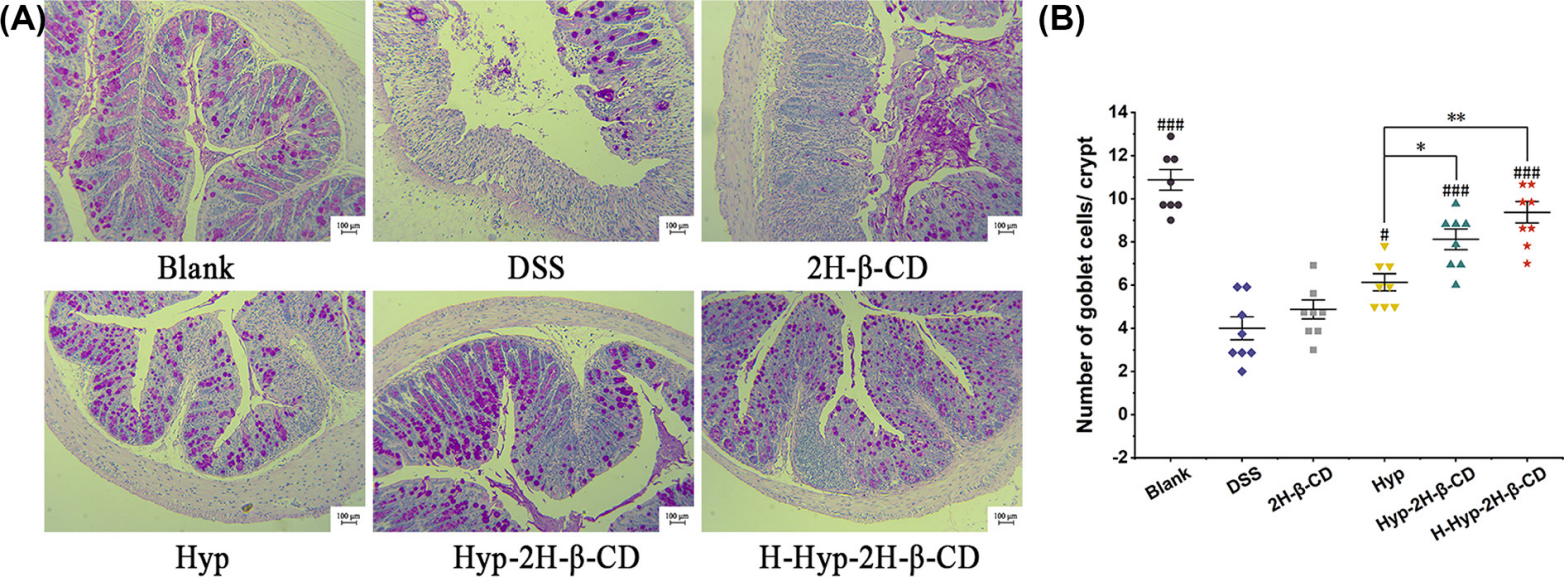
Effect of Hyp-2H-β-CD on goblet cells of colitis mice (**A**) Colonic histopathological sections of mice from each group (PAS staining, ×200 magnification). (**B**) Quantity changes of goblet cells in each group of mice's intestines. Note: In Figure (B), a comparison between two groups, *P<0.05; **P<0.01; compared with DSS group, ^#^*P*<0.05; ^###^*P*<0.0001.

Goblet cells are a kind of polarized columnar epithelial cells in the shape of a ‘goblet’, which are the main line of defense to form the intestinal mucosal barrier [36] and mainly secrete Mucoprotein 2 (MUC2) [37]. In the active stage of colitis, goblet cells decrease or even disappear, and their secretion function decreases [38]. This leads to the thinning of the mucus layer and increased intestinal permeability. The damage to the intestinal mucus barrier makes it difficult to resist the invasion of pathogenic bacteria [39]. In the study, PAS staining was used to count the goblet cells in the crypts. As shown in Figure 7A, goblet cells of the midgut mucosal epithelium in the blank group were stained fuchsia (PAS staining positive), arranged in an orderly manner, and mostly in the mature secretory state. Comparing the DSS group to the blank group, the DSS group’s crypt structure was damaged, the volume of goblet cells was decreased, and the number of goblet cells was dramatically decreased (*P*<0.0001), shown in Figure 7B. It indicated that DSS could directly destroy the intestinal mucosal barrier. The Hyp group had more goblet cells in their crypts than the DSS group (*P*<0.05), but Hyp-2H-β-CD could significantly boosted the number of goblet cells per crypt. At the same dose, the Hyp-2H-β-CD group and the Hyp group had significantly different goblet cell densities (*P*<0.05). The goblet cells were orderly arranged on both sides of the gland, and the crypt morphology was intact, indicating that Hyp-2H-β-CD had a stronger effect on restoring intestinal mucosal barrier function in mice.

#### Effect of Hyp-2H-β-CD on intestinal flora of mice

##### Operational Taxonomic Unit species

**Figure 8:**
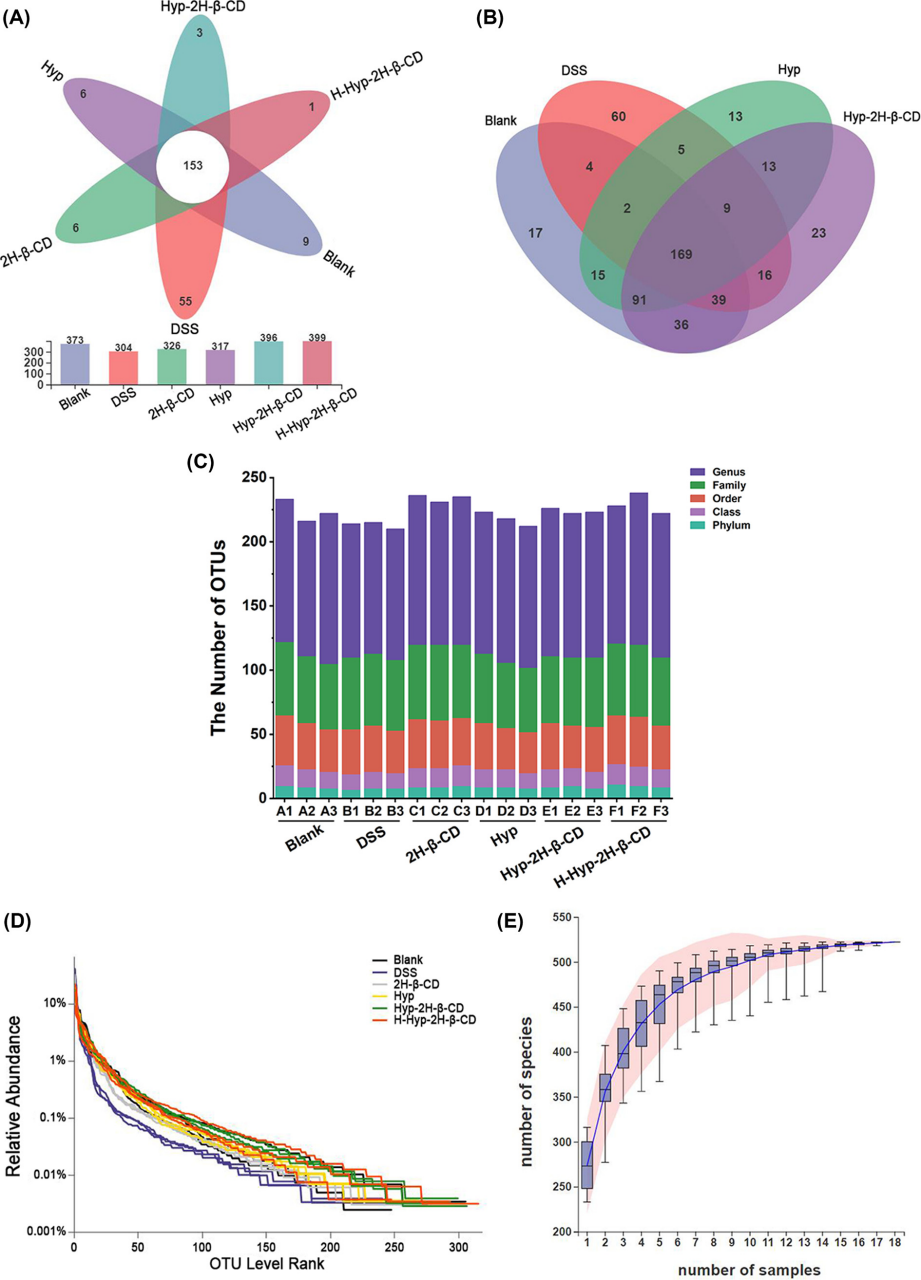
The analysis of the six experimental groups at OTU level (**A**) Flower petal diagram; (**B**) Venn diagram of OTU distribution in Blank group, DSS group, Hyp group, and Hyp-2H-β-CD group; (**C**) OTU types at five levels for each sample; (**D**) Rank-Abundance curve; (**E**) Species cumulative curve.

Among the 6 groups of mouse fecal samples, 18 samples generated a total of 82,8311 high-quality 16S rDNA sequences. All sequences were classified into operational taxon units (OTU) at a similar level of 97% following the same sequencing depth resampling of each sample. Following that, statistics on biological data were run. There were 522 OTUs in total, 11 phyla, 17 classes, 50 orders, 82 families, and 166 genera. The OTU species of each group varied from 304 to 399. Figure 8A clearly shows the number of unique and common OTUs in the intestinal tracts of different groups of mice, among which 153 common OTUs were found. Compared with the blank group, only 304 OTUs were found in the DSS group, but 55 were unique. The gut flora of the DSS group of mice was thought to be out of balance. In addition, there were 9 OTUs in blank group, 6 OTUs in 2H-β-CD group, 6 OTUs in Hyp group, 3 OTUs in Hyp-2h-β-CD group, and 1 OTUs in H-Hyp-2H-β-CD group. The OTU of the blank group, DSS group, Hyp group, and Hyp-2H-β-CD group in Venn Figure 8B were compared. There were 214 identical OTU between the blank group and the DSS group, 277 identical OTU between the blank group and the Hyp group, and 335 identical OTU between the blank group and the Hyp-2H-β-CD group. As a result, compared with the DSS group, the OTU species of mice in the Hyp group and Hyp-2H-β-CD group were nearer to those in the blank group, suggesting that Hyp-2H-β-CD could more successfully regulate the intestinal flora of colitis mice to normalize. Bar Figure 8C was created to graphically depict the differences in the OTU number and taxonomic status identification findings of each group. It was discovered that the drug group’s species richness was higher than that of the DSS group at all levels. The Rank-Abundance curve of all samples at the OTU level was drawn, as shown in Figure 8D. It was found that all sample curves tended to be balanced. It was indicated that at the level of this test area, microorganisms in the samples were captured in large quantities, and the test results were credible. Moreover, the rapid and steep decline of the curves of the DSS group and 2H-β-CD group indicated that the diversity of flora in the samples was low. The curves of the other groups were long and smooth, indicating that the species richness of the samples was high, the proportion of dominant bacteria was large, and the distribution was uniform. The cumulative curve is shown in Figure 8E, in which the number of OTUs increased with the number of samples extracted growing. There was a positive correlation between them. The cumulative curve eventually stabilized as the number of analyzed samples increased. The number of samples used in this investigation was adequate and reasonable.

##### Alpha analysis

**Figure 9:**
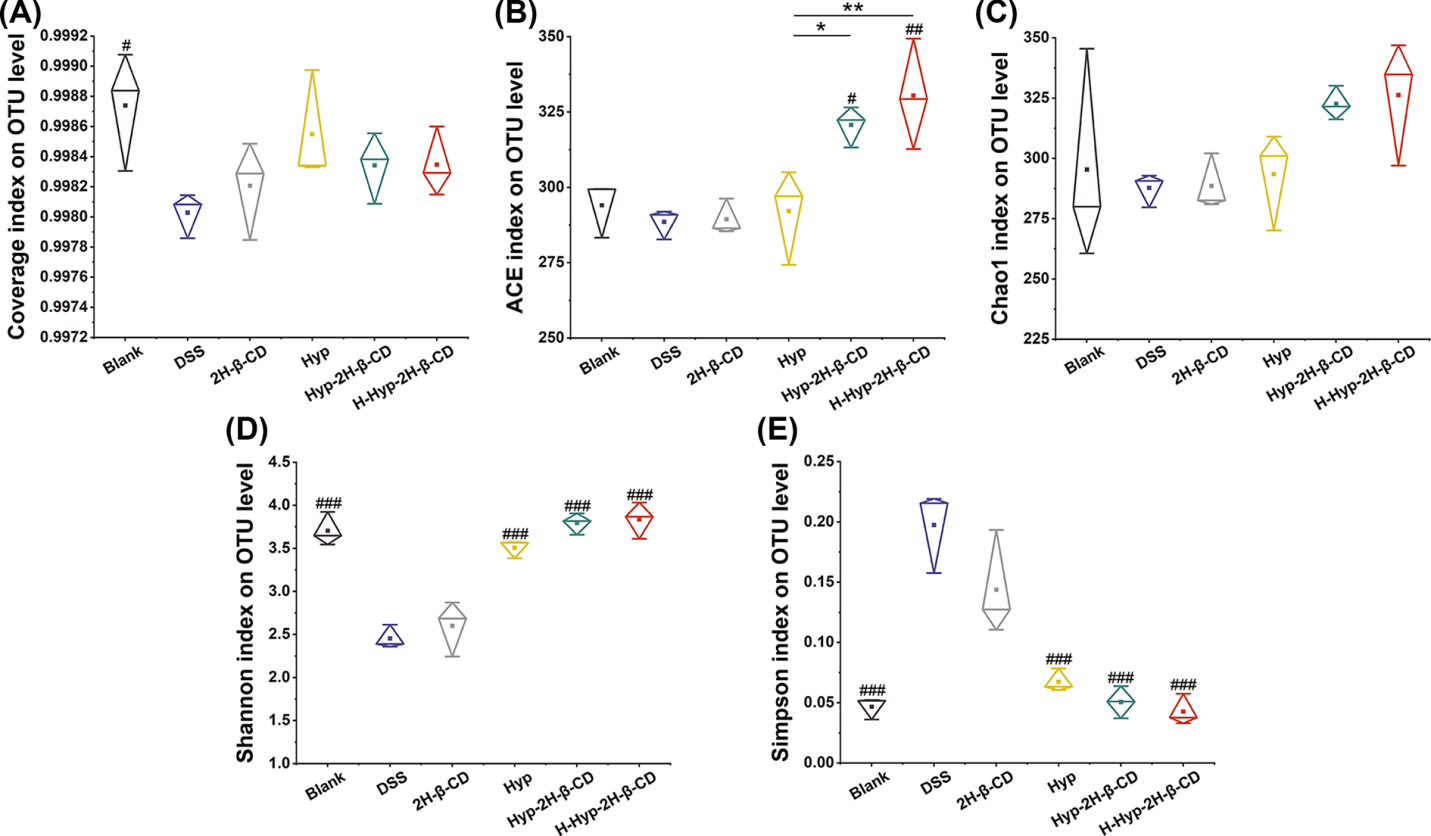
Based on the OTU level, the alpha diversity index data of the gut microbiota in each group (**A–E**) Coverage, ACE, Chao1, Shannon, and Simpson index. Note: In the figure (A**–**E), a comparison between two groups, *P<0.05; **P<0.01; compared with DSS group, ^#^*P*<0.05; ^##^*P*<0.01; ^###^*P*<0.0001.

The diversity within a given area or ecosystem is reflected by alpha diversity, a thorough indication of richness and evenness [40]. In this study, the flora richness of each group was assessed using the ACE index and the Chao1 index [41]. The flora diversity of each group was compared using the Shannon and Simpson indexes [40]. To determine if the sequencing findings accurately reflected the state of the microorganisms in the sample, coverage values were utilized, and the results are shown in Figure 9. In Figure 9A, the coverage of each group was greater than 0.99, indicating that the probability of sequence not being measured in the sample was extremely low. More than 99% of bacteria in all samples may have been captured, and the results were accurate and credible. In Figure 9B, the medication group’s gut microbiota ACE index rose compared with the DSS group. Only the Hyp group did not experience a significant rise (*P*>0.05), and the H-Hyp-2H-β-CD group and the Hyp-2H-β-CD group both did (*P*<0.05 and *P*<0.01). The mice’s ACE index was considerably greater than that of the Hyp group. And the values of the Chao1 index grew with time and were higher in the Hyp group, Hyp-2H-β-CD group, and H-Hyp-2H-β-CD group than in the DSS group. However, they did not differ significantly from one another (*P*>0.05), as shown in Figure 9C. These findings suggested that while Hyp could increase the richness of intestinal flora in mice, DSS could decrease its richness. Additionally, the synergistic impact improved with the addition of 2H-β-CD. Figure 9D clearly shows the Simpson index was significantly greater than the blank group and each drug group. In contrast, the Shannon index of the DSS group was significantly lower than the blank group and each drug group (*P*<0.0001) in Figure 9E. It was suggested that DSS induction would lower the species variety of the mouse digestive tract and cause an unequal species distribution. While Hyp and Hyp-2H-β-CD might repair and enhance the variety of the intestinal flora in DSS-induced colitis mice. Furthermore, each index's findings in the 2H-β-CD group were comparable to those in the DSS group, showing that 2H-β-CD by itself had no discernible impact on the variety of species in the injured gut.

##### Beta analysis

**Figure 10:**
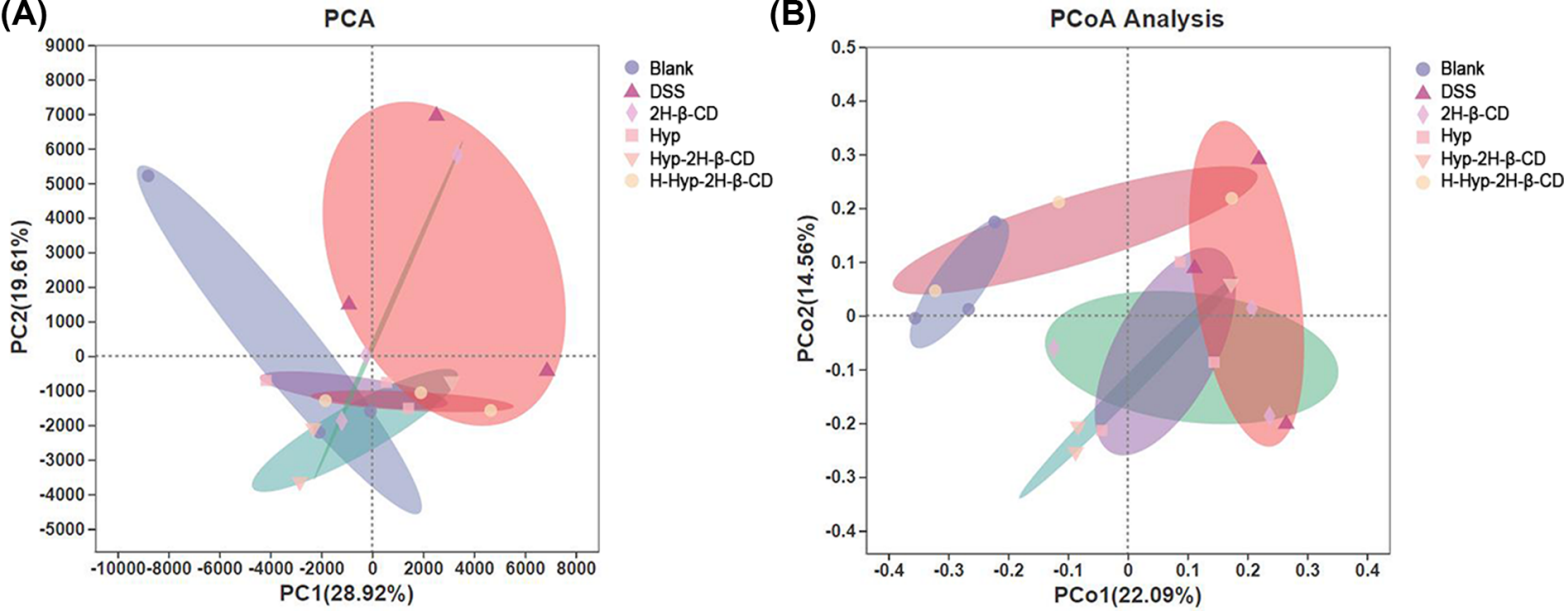
Beta diversity analysis of intestinal microbiota in each group based on OTU level (**A**) PCA analysis; (**B**) PCoA analysis.

To assess the similarity of intestinal microbiota structure between several mouse groups, principal component analysis (PCA) and principal coordinate analysis (PCoA) were performed [42]. The results are shown in Figure 10. In Figure 10A, the interpretation degrees of the PC1 axis and PC2 axes were 28.92% and 19.61%, respectively. In Figure 10B, the interpretation degrees of PCo1 and PCo2 to the differences in sample composition were 22.09% and 14.56%, respectively. It was discovered by PCA analysis that the six groups of sample composition varied significantly from one another. While the coordinates of the samples in the other groups demonstrated aggregation within the group, the coordinates of the DSS group and the 2H-β-CD group were not grouped and scattered. The blank group and the DSS group's confidence circles were the furthest apart. It was established that the DSS group and the blank group had the highest differences in the flora’s composition. Intestinal flora dysregulation was brought on in mice by the DSS challenge. In Figure 10B, the samples in each drug group were more concentrated and closer to the coordinates of the blank group. The coincidence degree of confidence circle between the H-Hyp-2H-β-CD group and the blank group was the highest. It was found that a high dose of Hyp-2H-β-CD might, to a certain extent, lessen individual variations between the groups and the DSS group, causing the damaged intestinal flora structure to more closely resemble that of a healthy individual.

##### Hyp-2H-β-CD regulates the taxonomic composition of intestinal flora

**Figure 11:**
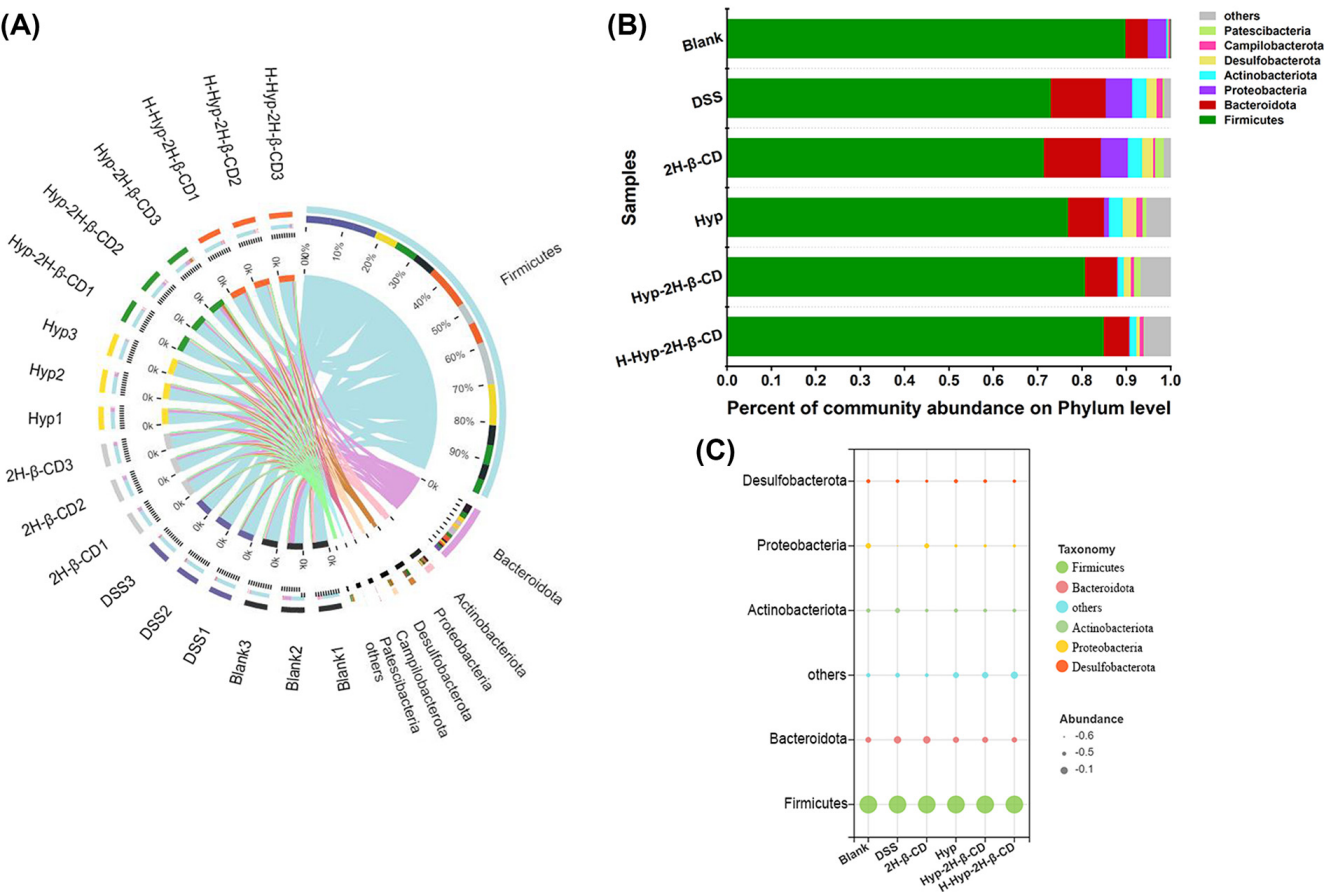
Analysis of intestinal microbiota composition based on phylum level in each group of mice (**A**) Circos diagram; (**B**) Bar analysis chart of the top seven dominant phyla; (**C**) Bubble chart of the relative richness of the top five bacteria at the phylum level.

Through taxonomic analysis, a total of 11 phyla were identified. In Figure 11A of the relationship between Circo’s samples and species, it was found that the composition of the dominant phyla in each sample was similar but with different proportions. The two most prevalent phyla were Firmicutes and Bacteroidota, with Proteobacteria and Actinobacteriota coming in second. Other phyla, such as Desulfobacterota, Campilobacterota, and Patescibacteria, which were less abundant, have also been detected. Many members of Firmicutes were beneficial bacteria that, through encouraging the release of anti-inflammatory mediators, control the inflammatory response. As successful competitors in the intestinal ecosystem, it was impossible to judge the absolute negative or positive impact of Bacteroidota on the host, but there were many pathogenic Bacteroides. Additionally, there was a positive correlation between the proportions of proteobacteria and actinobacteria and the level of inflammation. Figure 11B displays the composition and structural analyses of each bacterial phylum. The average proportion of Firmicutes in the blank group, DSS group, 2H-β-CD group, Hyp group, Hyp-2H-β-CD group, and H-Hyp-2H-β-CD group was 89.90%, 73.04%, 71.54%, 76.95%, 80.78%, and 85.02%, respectively. The average proportion of Bacteroidota was 5.00%, 12.40%, 12.81%, 8.13%, 7.19% and 5.69% respectively. In addition, as shown in bubble Figure 11C, the proportions of Proteobacteria and Actinobacteria in the DSS group and 2H-β-CD group increased significantly compared with those in the control group. In contrast, it decreased successively in the Hyp group, Hyp-2H-β-CD group, and H-Hyp-2H-β-CD group. These results indicated that both Hyp and Hyp-2H-β-CD could change the composition of the microbial population, and Hyp-2H-β-CD could better normalize the damaged bacterial flora.

**Figure 12:**
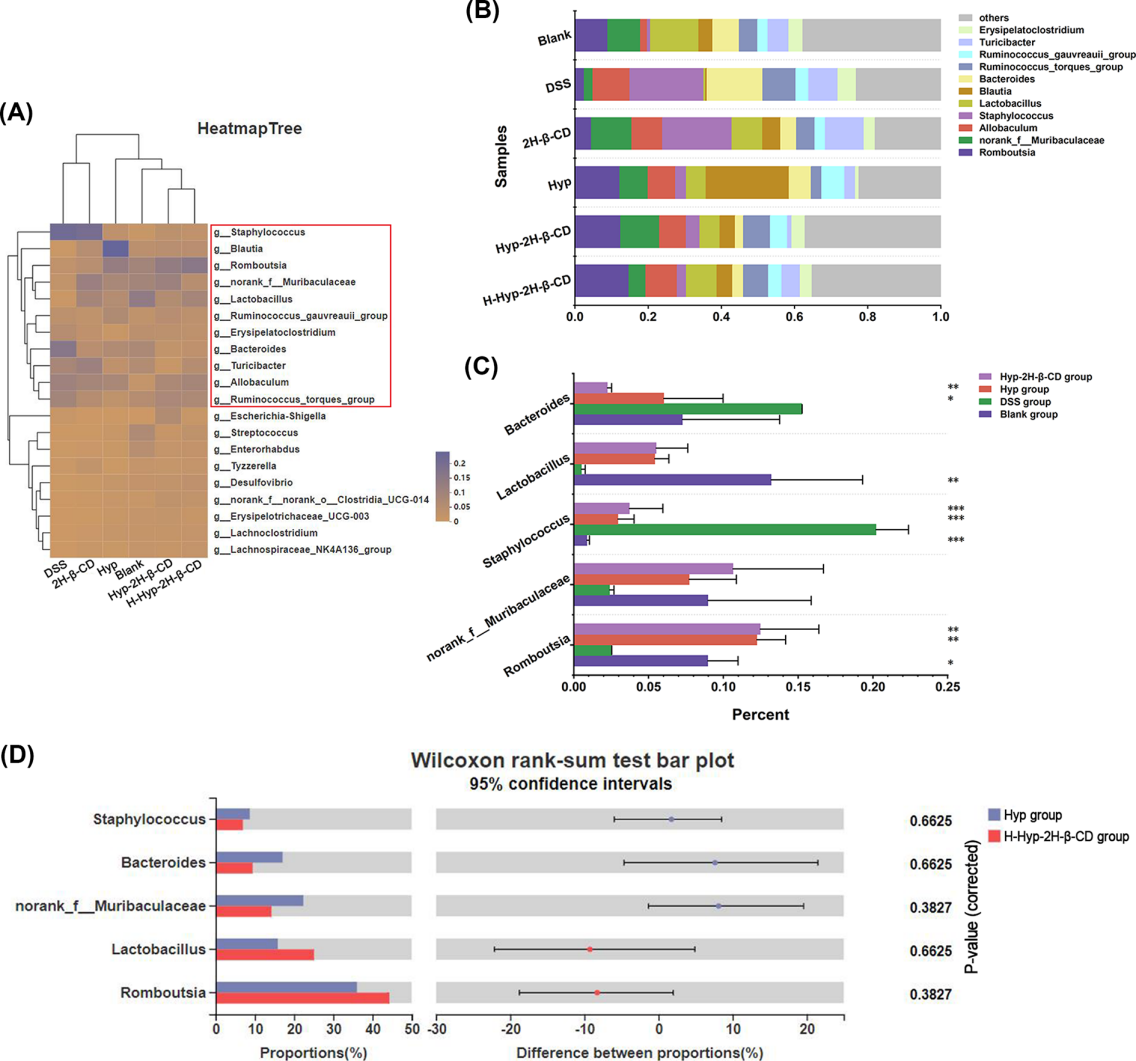
Analysis of intestinal microbiota composition in mice at the genus level (**A**) A heat map with the phylogenetic tree. (**B**) Bar analysis chart of the relative richness of the top 11 dominant bacterial genera. (**C**) Differences chart in the relative richness of five dominant bacterial genera among Blank group, DSS group, Hyp group, and Hyp-2H-β-CD group; (**D**) Histogram of confidence intervals for difference test between Hyp group and Hyp-2H-β-CD group. Note: In Figure (**C**), compared with the DSS group, *P<0.05; **P<0.01; ***P<0.0001.

To further verify the taxonomic differences, heat map Figure 12A was used to show the abundance changes of different genera in the samples. According to the results, the top 11 species with richness were characterized, and it was found that Romboutsia and norank_f_Muribaculaceae accounted for a large proportion in the blank group. Staphylococcus and Ruminococcus_torques_group accounted for a large proportion of the DSS group and 2H-β-CD group, similar to the intestinal biopsy results of Ulcerative Colitis patients conducted by Sokol et al. [43]. In addition, as the dose of Hyp-2H-β-CD increased, the intestinal microbiota structure of mice was close to that of normal mice, as shown in Figure 12B. Analysis was done on the variations between the five main bacterial taxa. Figure 12C presents the findings that Romboutsia (*P*<0.05), Lactobacillus (*P*<0.01), and other helpful bacteria were considerably reduced in the fecal samples as compared to the control group. While the relative richness of dangerous bacteria, such as Staphylococcus and others, dramatically increased (*P*<0.0001). Without a discernible difference (*P*>0.05), the proportions of Bacteroides grew, and norank_f__Muribaculaceae dropped. After treatment with Hyp and Hyp-2H-β-CD, the above bacterial genera showed the opposite trend compared with the DSS group. Staphylococcus was present in much lower amounts than in the DSS group (*P*<0.0001). Hyp-2H-β-CD had a better impact. The significance of differences between the Hyp group and the H-Hyp-2H-β-CD group was examined using the Wilcoxon rank-sum test. Figure 12D presents the findings of the amount of Romboutsia and Lactobacillus in the intestinal microbiota of mice in the H-Hyp-2H-β-CD group increased compared with the Hyp group. Staphylococcus and Bacteroides were less common but showed no significant changes (*P*>0.05). It was worth noting that Hyp-2H-β-CD could restore the proportion of norank_f__Muribaculaceae to normal, but it did not play a better effect when the dose was too large. This conclusion was limited to this genus. These results indicated that Hyp-2H-β-CD had a significant impact on the structure of the phylum-level composition of the gut flora and genus level in mice. Its ability to regulate the balance of flora was stronger than that of unincorporated Hyp.

## Conclusions

In the present study, we assessed the pharmacokinetics characteristics of Hyp-2H-β-CD in rats. The results showed that Hyp-2H-β-CD had higher drug blood concentrations. It was demonstrated that Hyp complexed with 2H-β-CD could improve its drug blood concentration and increases drug bioavailability, suggesting better clinical therapeutic effects. Therefore, we built a mouse model of DSS-induced colitis and contrasted Hyp before and after complexing therapeutic efficacy and mechanism. The investigation revealed that Hyp-2H-β-CD had better efficacy at the same dose than that of Hyp, which was in line with what was anticipated, both in terms of CMDI score and colonic lesions. This was consistent with previous studies indicating that Hyp complexed with 2H-β-CD had higher solubility and shorter dissolution time [20]. 16S intestinal microbiota structure analysis showed that Hyp-2H-β-CD could increase the abundance of beneficial bacteria and decrease the proportion of harmful bacteria to change the structural balance of intestinal microbiota in DSS-induced mice. It might provide therapeutic foundations for DSS-induced colitis mice.

## Data Availability

The data used to support the result of this study can be obtained from the corresponding author.

## Abbreviations

2H-β-CD: 2-hydroxypropyl-β-cyclodextrin
CD: crypt depth
DSS: dextran sodium sulfate
HPLC: high-performance liquid chromatography
OTU: operational taxon units
PCA: principal component analysis
PCoA: principal coordinate analysis
VH: villus height
